# Assessment of Primary Care Physicians’ Perception of Telemedicine Use During the COVID-19 Pandemic in Primary Health Care Corporation, Qatar

**DOI:** 10.7759/cureus.32084

**Published:** 2022-11-30

**Authors:** Khalid L Khan, Suleman Kanani, Mehr Nisa

**Affiliations:** 1 Family Medicine, Primary Health Care Corporation, Doha, QAT

**Keywords:** qatar, primary care, pandemic, covid-19, telemedicine

## Abstract

While telemedicine has been extensively researched throughout the globe, the Middle East has seen relatively little research on the topic. The purpose of this study was to investigate the primary care physicians' perceptions of the use of telemedicine, as well as its hurdles and benefits, during the COVID-19 pandemic in the state of Qatar.

In this multicenter cross-sectional study, an internally validated questionnaire was distributed among primary care physicians utilizing telemedicine during the pandemic at Primary Health Care Corporation (PHCC), the main primary care provider in Qatar. A convenience sample was taken due to the pandemic restrictions.

Out of 254 analyzed questionnaires, about half of the physicians (48%) had used telemedicine in the past primarily in the form of telephone consultations. Nearly three-quarters (74%) of physicians agreed that telemedicine is an easy way to communicate during the pandemic, but only half (52%) felt it improved job performance and effectiveness. Most physicians (90%) agreed that telemedicine is safe during the pandemic, but opinion was split on whether an optimum assessment of COVID-19 disease could be conducted. The majority also considered telemedicine an effective tool for chronic disease reviews (63%) and other consultations such as blood test results and medicine prescriptions (71%). The most significant barrier to telemedicine use was a language barrier followed by a lack of proper training. About 79% of responders felt that telemedicine improves access to healthcare and assists in contacting difficult-to-reach individuals, and 74% also thought it minimizes no-shows in the clinics.

Our study has shown that most physicians felt comfortable and safer using telemedicine as an alternative means to conduct consultations during the pandemic. Keeping in view its advantages, the majority suggested that it could be incorporated into daily practice even beyond the pandemic. However, concerns were raised about its use to assess COVID-19 disease, lack of training, and potential barriers such as language differences. Further studies are needed to assess the efficiency and cost-effectiveness of telemedicine as well as the evaluation of alternative media such as video consultation, which could increase the utility of telemedicine and potentially mitigate some of its disadvantages.

## Introduction

In November 2019, a novel coronavirus disease, COVID-19, was detected in Wuhan, China, which swiftly spread across the world, prompting the World Health Organization (WHO) to declare a global health emergency [1]. The pandemic triggered many countries to impose nationwide population lockdowns and implement policies like social distancing and reduced contact time between individuals in order to limit the spread of COVID-19. These lockdowns and policies had significant impacts on daily life as well as most healthcare systems across the world, which were confronted with not just the treatment of infected patients but also routine patient care.

The pandemic led us to focus our attention on models of healthcare delivery that limit face-to-face interaction between healthcare professionals and patients to minimize the spread of COVID-19. Like many countries, Qatar also initiated new remote healthcare services using telephone and video consultations. This method of care is generally referred to as telemedicine. The WHO defines telemedicine as “the delivery of health care services, where distance is a critical factor, by all health care professionals using information and communication technologies for the exchange of valid information for the diagnosis, treatment, and prevention of disease and injuries … in the interests of advancing the health of individuals and their communities” [2].

The use of telemedicine is well documented in the medical response to natural disasters [3,4]. In the wake of COVID-19, there has been a rise in the use of telemedicine for outpatient and inpatient care, including triage of possible COVID-19 cases. Healthcare professionals across the globe now routinely use telemedicine interventions as a method of conducting consultations. According to Corbett et al., telemedicine can be beneficial, particularly in the treatment of chronic disorders [5]. Telemedicine has been shown to improve the efficiency and quality of services and patient care while reducing costs and workload when compared to traditional face-to-face clinic-based delivery of care [6-8]. The addition of video to telephone consultations has been found to result in fewer medication errors, greater diagnostic accuracy, and improved decision-making [9]. Even before the pandemic, telemedicine services were a part of the national digital health strategy of Qatar’s National Health Vision 2018-2022 [10].

Our study aimed to analyze the physicians’ perspectives on the use of telemedicine in primary care since it has been established that user satisfaction is critical in the acceptance and adoption of telemedicine [11]. The views of physicians on the use of telemedicine have been studied throughout the world, but studies in the Middle East are sparse. A systematic review by Kruse et al. showed that barriers to the adoption of telemedicine differ greatly between countries [12]. Hence, research carried out in other countries is unlikely to be applicable to Qatar. Furthermore, most studies examining the utilization of telemedicine have been conducted in a secondary or tertiary care setting. These results, therefore, cannot be applied to primary care where workload, clinician tasks, and patient journeys differ considerably. Finally, even though telemedicine has been in use for some time, its rapid deployment across the globe due to COVID-19 is a new phenomenon. The widespread adoption of its usage allows for more precise statistical information about the perceived benefits and challenges. Moreover, the attitude toward its use for the assessment of patients with COVID-19 infection remains to be established. The utilization of telemedicine services in clinical settings is dependent upon the satisfaction of physicians and patients with the service [11]. As a result, assessing physicians’ perspectives on its implementation can assist to guarantee that telemedicine is used to its full potential. This study aims to bridge these gaps in the literature by evaluating an under-represented sample.

## Materials and methods

This was a descriptive cross-sectional study based on a questionnaire designed for clinicians utilizing telemedicine. The study was conducted at all 27 health centers working under the umbrella of Primary Health Care Corporation (PHCC), Qatar - the country’s main primary care provider. Due to COVID-19 pandemic constraints, a convenience sample size was used. The study's inclusion criteria were primary care and specialty physicians employed by PHCC in Qatar who used telemedicine services during the COVID-19 pandemic. Physicians who were not actively using telemedicine as well as other non-physician members of staff were excluded.

A questionnaire was formulated following a literature review identifying the key determinants of physicians’ attitudes to telemedicine [11]. Demographic information such as age, gender, and department was included for sub-group analysis, and questions about exposure to telemedicine use and types followed. Thereafter, a variety of statements regarding telemedicine were evaluated using a five-point Likert scale ranging from "strongly disagree" to "strongly agree." The statements were a mixture of positive and negative viewpoints on telemedicine use and categorized under the titles: perceived ease of use, perceived usefulness, barriers, and advantages.

A pilot study was conducted among 11 physicians across a variety of backgrounds based in one health center to assess the suitability and understandability of the questionnaire. The participants responded via an anonymized feedback form consisting of eight questions. The questionnaire was found to be of suitable length with acceptable timings of completion, with good clarity, structure, and format and without any perceived difficulties in completion or language. Two questions were modified based on the feedback received.

Ethics committee approval was obtained from the PHCC Research Sub-committee (PHCC/DR/2020/07/089), and all patients provided written informed consent to participate before data collection. Participation was wholly voluntary and anonymous with no identifiable personal information.

Questionnaires were sent to all 27 health centers and the centralized call center of PHCC via electronic and printed means. Responses were only received from 16 health centers and the call center. Completed questionnaires were received either manually or by internal post, and the results were manually entered onto an Excel sheet, with the labeling of the questionnaires for cross-checking. Descriptive statistics were used in the form of frequencies and percentages for data analysis.

## Results

Demographics and past use of telemedicine

In total, 264 questionnaires were received with a response rate of 39.9%. Only completed questionnaires were included in the analysis, reducing the sample size to 254. Table 1 outlines the demographics of the sampled population. Out of the 254 respondents, 154 (61%) were male and 100 (39%) were female. Nearly half of the respondents (47%) were between the ages of 41 and 50, with 24% between the ages of 31 and 40 and 16% between the ages of 51 and 60. The majority were family medicine physicians 215 (85%), with the remainder being from other specialties such as ENT, dermatology, and pediatrics working in primary care. Around half (48%) of the respondents had previous experience of using telemedicine prior to the COVID-19 pandemic. All the respondents who had previously utilized telemedicine were under the age of 50. Previous telemedicine experience was mainly through telephone consultations with only 17 participants having used video and even to a lesser degree other modalities such as emails (6). Interestingly, over two-thirds (69%) of females had not used any form of telemedicine services before the pandemic.

**Table 1:** Demographics and past use of telemedicine

		Number	Percentage	
Gender				
	Male	154	61%	
	Female	100	39%	
Age (years)				
	21-30	6	2%	
	31-40	61	24%	
	41-50	119	47%	
	51-60	43	17%	
	>61	26	10%	
Department				
	Family medicine	215	85	
	Others	39	15	
Past use of telemedicine				
	Total	121	48%	
	Male	84	69%	
	Female	37	31%	
No use of telemedicine				
	Total	132	52%	
	Male	69	52%	
	Female	63	48%	

Perceived ease of use of telemedicine

**Figure 1:**
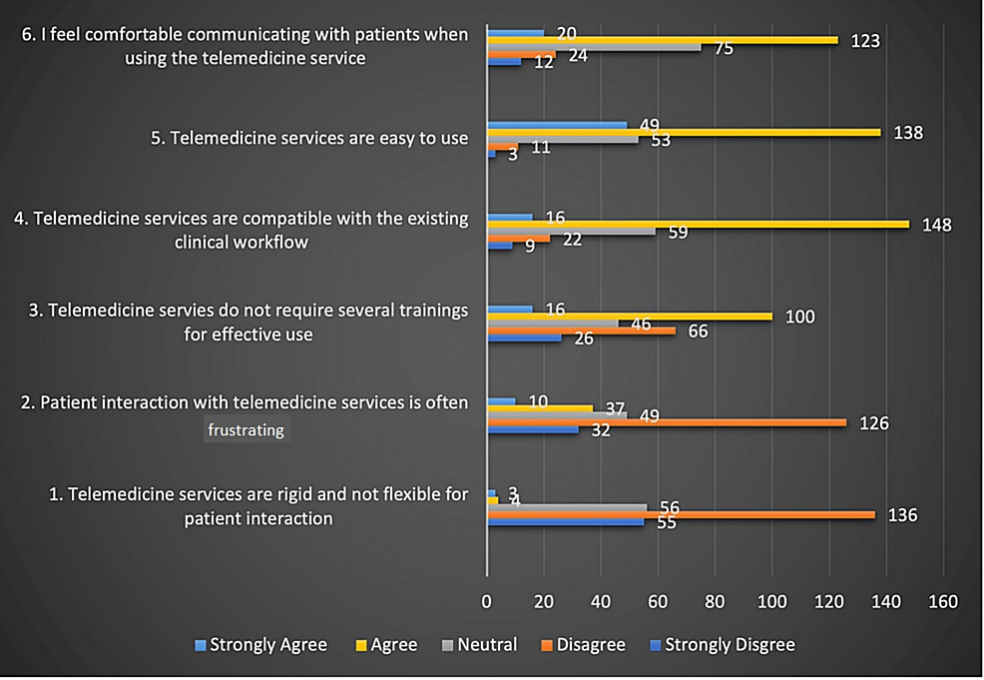
Perceived ease of use of telemedicine

As detailed in Figure 1, 157 (62%) disagreed or strongly disagreed with the notion that telemedicine services are rigid and inflexible for patient interaction; a similar percentage also thought that patient interaction was not frustrating via telemedicine. A total of 116 (46%) of the physicians agreed or strongly agreed that effective use of telemedicine would require several training sessions, whereas 92 (36%) disagreed or strongly disagreed with that. The majority of the respondents perceived telemedicine to be compatible with their existing clinical workflow (65%) and easy to use (74%), and 137 (65%) agreed or strongly agreed that they felt comfortable communicating with patients using telemedicine.

Perception of the usefulness of telemedicine in the assessment of COVID-19

**Figure 2:**
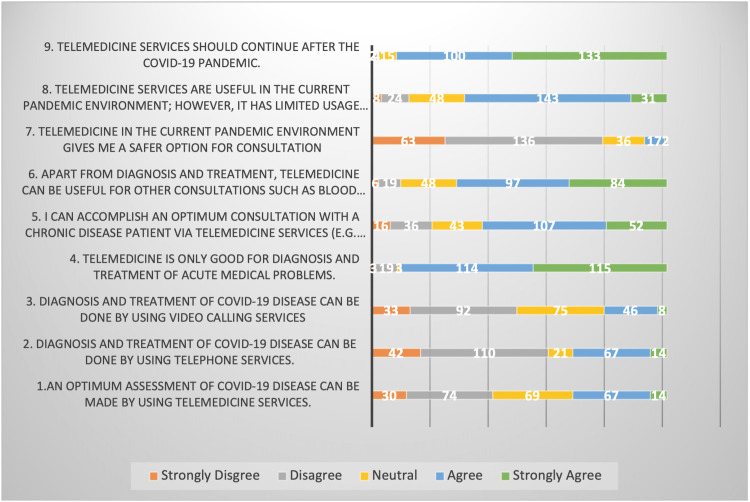
Perceived usefulness of telemedicine

Opinion was split about whether telemedicine services can provide an optimum assessment of COVID-19 disease. Most respondents disagreed that an accurate evaluation could be performed using either telephone services (60%) or video consultations (49%). However, an overwhelming majority (90%) believed that telemedicine was a safer alternative in a pandemic situation. A significant number of the respondents (67%) felt it would be beneficial to keep using telemedicine services even after the pandemic is over. A majority of participants considered telemedicine beneficial not only for evaluating acute medical problems but also for chronic illness reviews such as diabetes (69%) and also for other routine work such as blood test results, as shown in Figure 2.

The pandemic triggered many countries to impose nationwide population lockdowns and implement policies like social distancing and reduced contact time between individuals in order to limit the spread of COVID-19. These lockdowns and policies, although deemed essential from a public health perspective, had a significant impact on our daily lives as well as most healthcare systems across the world, which were confronted with not just the treatment of infected patients but also routine patient care and prescription requests (92%).

Barriers to effective telemedicine use

**Figure 3:**
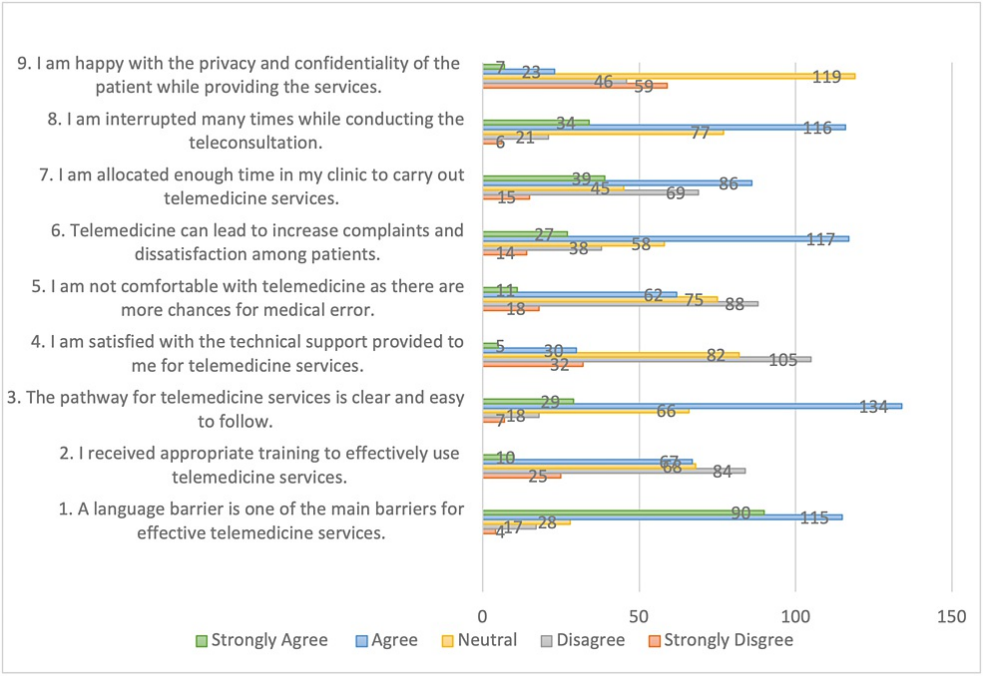
Barriers to effective telemedicine use

As seen in Figure 3, the most significant impediment to efficient telemedicine services was shown to be the language barrier (80%). Approximately two-thirds of respondents (64%) believed that the protocol for use of telemedicine was obvious and simple to follow. About 54% were pleased with the technical assistance they had received. Similarly, more than half were pleased with the amount of time allotted for telemedicine use (57%) and the preservation of a patient’s privacy/confidentiality (59%) during such encounters.

Only 30% of the physicians felt that adequate training for using telemedicine services was provided, with 43% disagreeing. While 29% thought telemedicine could lead to an increased risk of medical mistakes, 42% disagreed. Finally, a majority (49%) thought they were interrupted frequently during telemedicine consultations

Advantages of telemedicine

**Figure 4:**
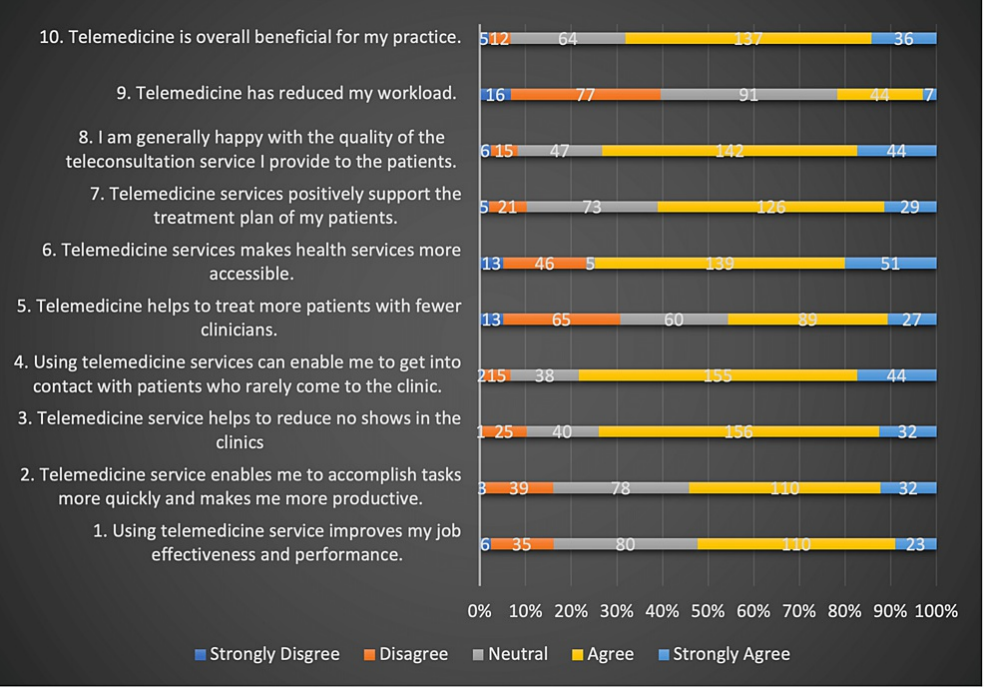
Advantages of telemedicine

Most participants (68%) saw telemedicine as advantageous to their practice, boosting work effectiveness (52%), and increasing productivity (53%). Most were happy with the quality of services they provided (73%), felt that telemedicine improves access to health care (79%), assists in contacting difficult-to-reach individuals (78%), and minimizes no-shows in clinics (74%). However, respondents were divided on whether telemedicine lessens the physician workload, with 37% disagreeing and 28% agreeing. About 46% of physicians also thought that an increased number of patients could be seen and assessed using telemedicine services. These results are detailed in Figure 4.

## Discussion

Overview of findings

Although telemedicine was first introduced in the late 19th century, its function and ramifications remained unclear for multiple decades [13]. Our findings highlight a paucity of usage of telemedicine in primary care in the state of Qatar prior to the COVID-19 pandemic, with less than half of the participants having any experience with this technology. Nonetheless, the COVID-19 pandemic accelerated the implementation of telemedicine, demonstrating its advantages and provided an opportunity to explore its limitations. We discovered that the majority of our respondents considered telemedicine as beneficial to their practice during the pandemic. Physicians faced several challenges including the perception that optimum assessment of COVID-19 illness was difficult via telephone or video consultations, as well as the difficulties of a language barrier between patients and physicians, and interruptions during teleconsultations. However, the benefits of using telemedicine in Qatar during the pandemic were felt to outweigh the challenges it brought.

Previous use and ease of use of telemedicine

Prior to the COVID-19 pandemic, less than half of the respondents, all under the age of 50, had used telemedicine. In sub-group analysis, individuals who had never used telemedicine before were more likely to believe that there was a higher risk of medical errors and that telemedicine might mean treating more patients with fewer physicians. These perceptions could be influenced by their inexperience in the use of telemedicine. We also observed that male respondents were more familiar with telemedicine prior to the pandemic than females, with two-thirds of females reporting they had not used any kind of telemedicine. This is corroborated by the literature showing higher adoption and intention to use telemedicine among males, although the reason for this remains unclear [14,15].

Furthermore, despite the availability of video consultations, our participants only used telephone consultations. Although the reasons for preferring audio over video consultations were not assessed in our study, video consults can result in increased physicians’ confidence in managing acute consultations and the ability to provide patient education [16]. Studies have shown that the primary variables influencing physicians' adoption of telemedicine are the convenience of use and knowledge of usefulness [17]. The hesitation in our cohort regarding its use requires further exploration.

Most of our participants believed that telemedicine was not inflexible, not frustrating, compatible with the existing workflow, and easy to use. Most participants did not believe that training sessions were necessary for optimal usage, although only one-third felt adequate training was provided. Telehealth’s effective implementation into routine care has been found to require appropriate systematic education and training for physicians [18]. The low perceived need for training might be owing to the relative simplicity of audio consultations utilized exclusively by our cohort as opposed to video consults that require some technical expertise.

Perceived usefulness of telemedicine services

All respondents agreed that neither telephone services nor video consultation could provide an accurate assessment of COVID-19 illness. Pre-pandemic, Haimi et al. investigated the experiences, attitudes, and obstacles of physicians in an Israeli Pediatric Telemedicine Service while making clinical decisions using telemedicine. Their findings emphasized seven key themes about the obstacles encountered when using this service, one of which was remote diagnostics. More than two-thirds of responders in their survey expressed worries about making a distant diagnosis and recommending medication without personally examining the patient [19]. However, studies during the pandemic, including ours, seem to agree that telemedicine is a safer and more suitable option for assessing patients during pandemic times, likely due to the rebalanced calculation of risk with closer physical contact [20].

Aside from COVID-19, our data indicated that telemedicine interventions were extremely beneficial for the evaluation of patients with chronic diseases and other non-urgent consultations, including diabetes reviews, blood test results, and medication refill requests. Corbett et al. substantiated these findings, highlighting that telemedicine has the potential to revolutionize the treatment of chronic disease [5]. Furthermore, AlAhmad et al. have received approval for a research protocol that will explore the influence of telemedicine on chronic disease management and follow-up compliance across three primary healthcare institutions in Qatar. The results of this study are to be published in late 2022 and will provide greater insight into the role of telemedicine in Qatar for the management of chronic diseases [21].

Barriers to effective telemedicine services

A language barrier was the most common impediment to telemedicine deployment among our respondents, followed by interruptions during patient consultations. These findings, although valid, are not corroborated by current literature. Kruse et al. conducted a systematic review that evaluated the barriers to adopting telemedicine worldwide. His findings identified 33 individual barriers, with the most prevalent challenges being technically challenged staff, resistance to change, cost, reimbursement, age of the patient, and level of education of the patient. A language barrier only consisted of 2% of all barriers, and frequent interruptions were not found at all in previous studies [12]. On the other hand, an additional review by Almathami et al. observed that both lack of body language and communication skills presented as a barrier to telemedicine adoption [22]. In other studies, clinicians have highlighted concerns regarding data security, training requirements, legal liability, time, workload, technical quality, the need for guidelines, and cultural challenges with regard to telemedicine [12,23-27]. Some of the hurdles found by previous studies may be unique to certain parts of the world or countries, so this needs to be kept in mind when comparing results. For instance, concerns regarding data security and legal issues observed in other studies as barriers to effective telemedicine deployment are typically not regarded as issues in Qatar or other Middle Eastern countries, given the healthcare structure and organization [23,24]. Additionally, the wide diversity and range of multicultural demographics in Qatar compared to other countries may highlight cultural issues such as language differences and norms in interrupting doctors in their clinic rooms. This emphasizes the significance of local follow-up studies to validate our findings.

Advantages of telemedicine

Telemedicine was widely seen as a useful complement to practice, with the potential to improve work performance and productivity. Most respondents were pleased with the level of service they provided in our study. When investigating telemedicine utilization and the viewpoints of referring primary care providers, Barton et al. discovered comparable results to show that while physicians who used telemedicine as part of their practice were aware of its limitations, they also recognized the great potential that it offered as a tool of providing consultation and care to their patients [28]. Telemedicine enhances access to health services, assists in contacting difficult-to-reach individuals, and minimizes clinic no-shows. Our findings corroborate with the literature where telemedicine has a high degree of satisfaction among physicians, and the majority are willing to use it [11,23-25,29]. More crucially, during a pandemic, telemedicine provides an alternative to traditional medicine, allowing clinical services to be delivered when face-to-face consults are not feasible, given the focus on containing a pandemic [30].

## Conclusions

Our data support the use of telemedicine as an alternative to traditional visits during pandemics. Respondents' perspectives and attitudes emphasize the physicians' desire to incorporate this technology into daily practice even beyond the pandemic. However, there are some restrictions and perception gaps that must be addressed, such as the necessity for training sessions to encourage video consultations and mitigate potential language barriers. Our study also can be useful to policymakers and stakeholders to address the barriers to utilization, which may streamline care and improve satisfaction. Further studies should focus on assessing the efficiency and cost-effectiveness of telemedicine and exploring themes identified such as specifying the distractions and language hurdles that practitioners experience at a local level. Finally, our study design only evaluated the opinions of physicians who are on one side of the consultation. Studies looking at the patient perspective on telemedicine use are crucial to ensure efficient and holistic care.

## Appendices

**Table 2:** Questionnaire

PART A – DEMOGRAPHICS										
Kindly provide general information about yourself by ticking the respective boxes or fill in the blank where applicable below:										
1. Age group (years):	□ <21	□21-30	□31-40		□41-50		□51-60			□ >61
2. Gender:	□ Male				□ Female					
3. Department:	□ Family/internal Medicine					□ Pediatrics			□ ENT	
	□ Ophthalmology					□ Dermatology			□ Other	
4. Have you used telemedicine services before this COVID outbreak for patient consultations?	□ Yes				□ No					
5. Which of the following telemedicine methods have you used before, if any?	□ Video conferencing			□Telephone				□ E-mailing		
	□ Mobile health monitoring devices						□ Other			
6. Which of the following telemedicine methods are you using now?	□ Video conferencing			□Telephone				□ E-mailing		
	□ Mobile health monitoring devices						□ Other			
PART B – VIEWS ON TELEMEDICINE										
Please indicate to what extent you agree or disagree with the following statements on physicians’ satisfaction with telemedicine services acceptance by ticking the appropriate number/scale. (0 = Not sure; 1 = Strongly Disagree; 2 = Disagree; 3 = Neutral; 4 = Agree; 5 = Strongly Agree)										
Perceived Ease of Use	0 - Not Sure		1 - Strongly Disagree	2 - Disagree	3 - Neutral		4 – Agree			5 - Strongly Agree
Telemedicine services are rigid and not flexible for patient interaction.										
Patient interaction with telemedicine services is often frustrating.										
Telemedicine services do not require several training for effective use.										
Telemedicine services are compatible with the existing clinical workflow.										
Telemedicine services are easy to use.										
I feel comfortable communicating with patients when using the telemedicine service.										
Perceived Usefulness of Telemedicine Services	0 - Not Sure		1 - Strongly Disagree	2 - Disagree	3 - Neutral		4 - Agree			5 - Strongly Agree
An optimum assessment of COVID-19 disease can be made by using telemedicine services.										
Diagnosis and treatment of COVID-19 disease can be done by using telephone services.										
Diagnosis and treatment of COVID-19 disease can be done by using video calling services.										
Telemedicine is only good for the diagnosis and treatment of acute medical problems.										
I can accomplish an optimum consultation with a chronic disease patient via telemedicine services (e.g., diabetic review).										
Apart from diagnosis and treatment, telemedicine can be useful for other consultations such as blood test results, medication requests, etc.										
Telemedicine in the current pandemic environment gives me a safer option for consultation.										
Telemedicine services are useful in the current pandemic environment; however, it has limited usage overall.										
Telemedicine services should continue after the COVID-19 pandemic.										
Barriers to Effective Telemedicine Services	0 - Not Sure		1 - Strongly Disagree	2 - Disagree	3 - Neutral		4 - Agree			5 - Strongly Agree
A language barrier is one of the main barriers to effective telemedicine services.										
I received appropriate training to effectively use telemedicine services.										
The pathway for telemedicine services is clear and easy to follow.										
I am satisfied with the technical support provided to me for telemedicine services.										
I am not comfortable with telemedicine as there are more chances for medical errors.										
Telemedicine can lead to increased complaints and dissatisfaction among patients.										
I am allocated enough time in my clinic to carry out telemedicine services.										
I am interrupted many times while conducting the teleconsultation.										
I am happy with the privacy and confidentiality of the patient while providing the services.										
Advantages of Telemedicine Services	0 - Not Sure		1 - Strongly Disagree	2 - Disagree	3 - Neutral		4 - Agree			5 - Strongly Agree
Using telemedicine service improves my job effectiveness and performance.										
Telemedicine service enables me to accomplish tasks more quickly and makes me more productive.										
Telemedicine service helps to reduce no- shows in the clinics.										
Using telemedicine services can enable me to get into contact with patients who seldom come to the clinic.										
Telemedicine helps to treat more patients with fewer clinicians.										
Telemedicine services makes health services more accessible.										
Telemedicine services positively support the treatment plan of my patients.										
I am generally happy with the quality of the teleconsultation service I provide to the patients.										
Telemedicine has reduced my workload.										
Telemedicine is overall beneficial for my practice.										
